# Molecular genetic diversity and population structure of Ethiopian white lupin landraces: Implications for breeding and conservation

**DOI:** 10.1371/journal.pone.0188696

**Published:** 2017-11-30

**Authors:** Mulugeta Atnaf, Nasser Yao, Kyalo Martina, Kifle Dagne, Dagne Wegary, Kassahun Tesfaye

**Affiliations:** 1 Pawe Research Center, Ethiopian Institute of Agricultural Research, Pawe, Ethiopia; 2 Biosciences eastern and central Africa- International Livestock Research Institute (BecA-ILRI) Hub, Nairobi Kenya; 3 Department of Microbial, Cellular and Molecular Biology, College of Natural Sciences, Addis Ababa University, Addis Ababa, Ethiopia; 4 CIMMYT-Ethiopia, ILRI Campus, CMC Road, Addis Ababa, Ethiopia; Università Politecnica delle Marche, ITALY

## Abstract

White lupin is one of the four economically important species of the *Lupinus* genus and is an important grain legume in the Ethiopian farming system. However, there has been limited research effort to characterize the Ethiopian white lupin landraces. Fifteen polymorphic simple sequence repeat (SSR) markers were used to assess the genetic diversity and population structure of 212 Ethiopian white lupin (*Lupinus albus*) landraces and two genotypes from different species (*Lupinus angustifolius* and *Lupinus mutabilis*) were used as out-group. The SSR markers revealed 108 different alleles, 98 of them from 212 landraces and 10 from out-group genotypes, with an average of 6.5 alleles per locus. The average gene diversity was 0.31. Twenty eight landraces harbored one or more private alleles from the total of 28 private alleles identified in the 212 white lupin accessions. Seventy-seven rare alleles with a frequency of less than 5% were identified and accounted for 78.6% of the total alleles detected. Analysis of molecular variance (AMOVA) showed that 92% of allelic diversity was attributed to individual accessions within populations while only 8% was distributed among populations. At 70% similarity level, the UPGMA dendrogram resulted in the formation of 13 clusters comprised of 2 to 136 landraces, with the out-group genotypes and five landraces remaining distinct and ungrouped. Population differentiation and genetic distance were relatively high between Gondar and Ethiopian white lupin populations collected by Australians. A model-based population structure analysis divided the white lupin landraces into two populations. All Ethiopian white lupin landrace populations, except most of the landraces collected by Australians (77%) and about 44% from Awi, were grouped together with significant admixtures. The study also suggested that 34 accessions, as core collections, were sufficient to retain 100% of SSR diversity. These accessions (core G-34) represent 16% of the whole 212 Ethiopian white lupin accessions and populations from West Gojam, Awi and Australian collections contributed more accessions to the core collection.

## Introduction

*Lupinus* is a large and diverse genus in family Fabaceae. The number of species belonging to this genus is not well defined and different published works mention a range from 200 to over 1700 [1–3]. However, there are only four species with agricultural importance, viz., *L*. *albus*, *L*. *angustifolius*, *L*. *luteus* and *L*. *mutabilis*. White lupin (*Lupinus albus*; 2n = 50) has been cultivated for several thousand years in the Mediterranean region where it originated, and along the Nile valley (including Ethiopia)[4]. It is widely known, commercially important, large seeded, annual lupin species in the world and is a promising annual legume crop for human consumption, green manuring and forage. White lupin has also substantial human nutrition and health importance, providing a number of functional food products [5–8].

In Ethiopia, white lupin exclusively grown by smallholder subsistence farmers and is valued for its grain and soil fertility maintenance properties[9]. Traditionally, it has been used to cure a number of disorders and diseases including high blood pressure. The crop also has social value, being generally consumed in bad years for the harvest of other crops and by poor communities. Furthermore, marketing of processed products and sometimes the grain itself is mainly carried out by women and youth [10].However, preparing food and beverages from white lupins involves long and laborious steps to get rid of the bitter taste, which is due to the presence of high level of alkaloids in Ethiopian white lupin local cultivars. Moreover, despite its importance to improve the fertility of degraded farm land and as an economical source of protein for poor families, only very limited research and development efforts have been made to improve lupin productivity and quality in Ethiopia, hence to date no single improved food variety has been availed to the farmers [9, 10].

For any improvement and conservation management strategy, understanding the genetic diversity between and within populations is an important step [11]. Major collections of important crop plants are held in gene banks around the world. These collections serve as repositories of the biodiversity available for each species and thus are a valuable source of genes useful to plant breeders. Nevertheless, the utility for breeding and conservation of these germplasms in gene bank collections depends largely on the accuracy of the evaluation data [12]. Furthermore, establishing a core collection, *i*.*e*., a group of accessions from an existing germplasm collection that is chosen to represent the genetic spectrum of the entire collection, could facilitate efficient use and management of large collections [13].

The genetic diversity of white lupin and other species of lupin conserved in different parts of the world has been characterized using morphological and agronomical attributes [14], isozymes/proteins [15] and molecular markers including random amplified DNA polymorphism, amplified fragment length polymorphism and inter simple sequence repeats [16].Assessment of genetic diversity on the basis of morphological traits is not very reliable, since it may be influenced by the environment coupled with a small number of traits with known inheritance. Molecular markers such as microsatellites (SSRs) are numerous and have the distinct advantage of being independent of climatic variables. Microsatellites have been used and found to be a good choice in genetic diversity, population structure and gene mapping studies of different legumes including soybean [17], mungbean [18], lentil [19], field pea [20], faba bean [21], chickpea [22] and white lupin [23].The use of molecular markers can also help to select accessions for establishing a core collection and its application has been reported in different crops such as dry bean [24–27] and rice [28].

The Ethiopian Biodiversity Institute (EBI) conserved more than 275 white lupin landrace accessions. A study by Raman *et al*. [23]claimed that Ethiopian landraces represent a unique gene pool, although they considered only 7 Ethiopian landraces. Furthermore, it has been reported that Ethiopian white lupin landraces harbor genes that offer resistance to the wide-spread and devastating fungal disease anthracnose [29], sources of traits for climate resilience including drought resistance, and proteoid/cluster roots development for phosphorus scavenging [30].However, except for passport data and some phenotypic [31] and limited ISSR [32] characterizations, these landrace accessions have never been systematically characterized using molecular markers.

Thus, the objectives of this study were: to characterize the genetic diversity of Ethiopian white lupin accessions using SSR markers, to determine the intra-and inter-population genetic diversity and to establish a core collection of Ethiopian white lupin.

## Materials and methods

### 2.1 Plant materials and DNA extraction

**Fig 1:**
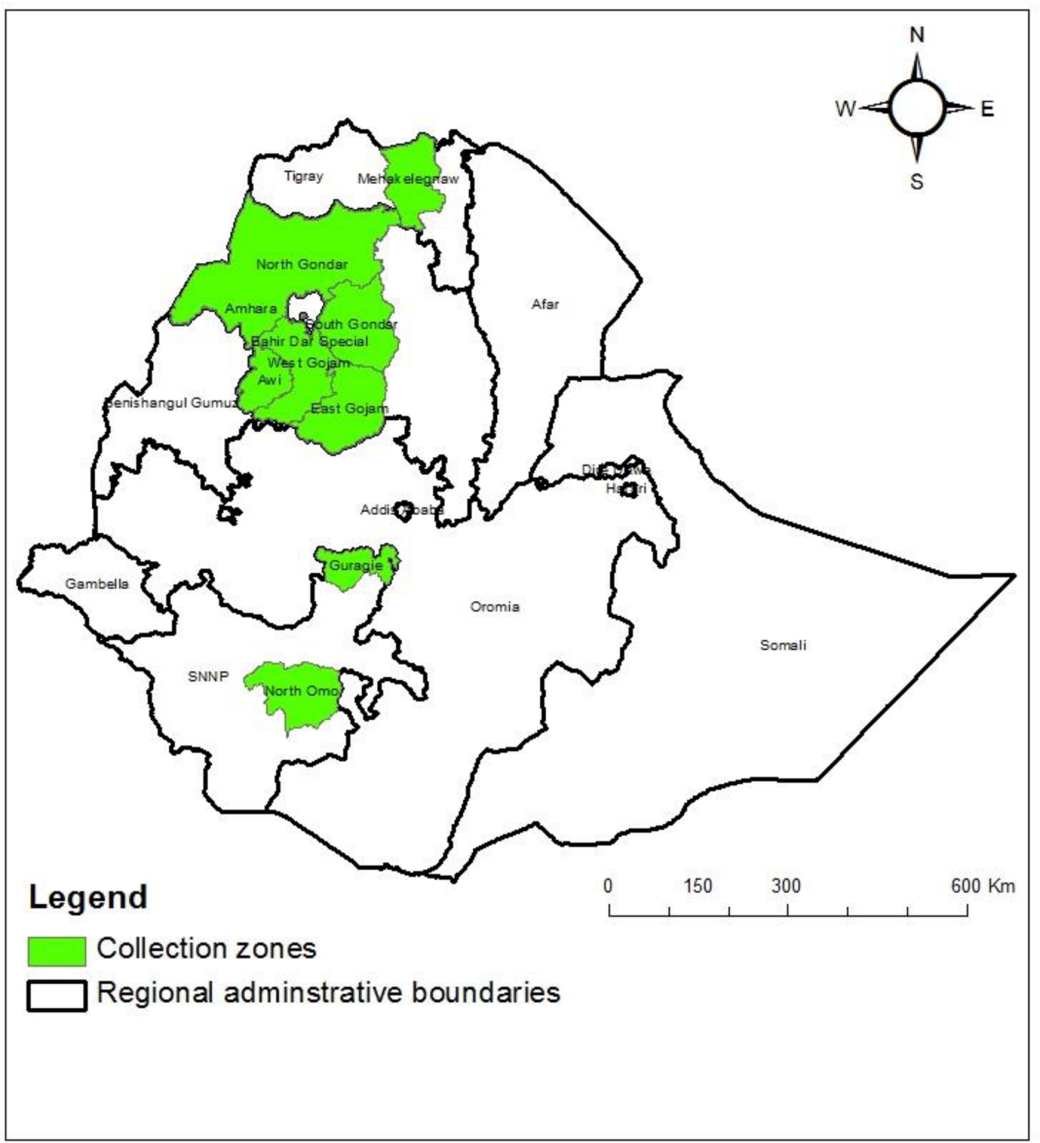
Map of collection areas of white lupin accessions which are used in the study. This map is “Reprinted from [33] under a CC BY license, with permission from [AJCS], original copyright [2017]”.

The 212 white lupin landrace accessions and one *Lupinus mutabilis* genotype used in this study were obtained from the Ethiopian Biodiversity Institute (EBI), whereas one *Lupinus angustifolius* genotype was kindly provided by Dr. Alemayehu Assefa (Researcher at Adet research center, Ethiopia). The landraces were mainly collected in northwestern Ethiopia (West Gojam, 87 accessions; East Gojam, 35 accessions; Awi, 38 accessions; and Gondar, 32 accessions) as revealed from the passport data. In addition, 20 white lupin accessions which are without passport data (believed to have been collected by Australians in Ethiopia and donated to the EBI) and the two genotypes each from different species used as out-group, were included in this study. We considered all the accessible (those that are in active collections) landrace accessions conserved in the gene bank of EBI and these number of sampled landraces represents 75% of the Ethiopian white lupin landrace accessions collected from all lupin producing zones of the country (Personal communication to EBI database officers). Full descriptions of these accessions and a map of their collection areas are provided in S1 Table and Fig 1.

A total of 178 accessions were grown in Ethiopia and sampled leaves were dried using silica gel and transported to the Biosciences eastern and central Africa-International Livestock Research Institute (BecA-ILRI)Hub in Nairobi, Kenya. The remaining 36 accessions were planted at the BecA-ILRI hub screen house, for DNA extraction. Approximately equal amounts of leaves from five different plants per accession were bulked to represent the accession [12]. Genomic DNA was extracted from bulked leaves of 15 days to one month old seedlings from each accession, using QIAGEN kit at the BecA-ILRI Hub. Genomic DNA quality was checked through 1%agarose gel electrophoresis and Thermo Scientific NanoDrop Spectrophotometer 2000c. The total amount of DNA was quantified with a NanoDrop Spectrophotometer and normalized to 10ng/μl for polymerase chain reaction (PCR) and genotyping.

### 2.2 PCR and SSR markers assay

For SSR assays, a total of 16 polymorphic SSR markers based on reports by Raman et al.[23] and Phan et al.[34] were selected (S2 Table). PCR amplification reactions were carried out on GeneAmp PCR system 9700 thermo cycler in a total reaction volume of 10 ml lyophilized negative dye BIONEER 96 well plate, 1.5–2.5 μL of genomic DNA template, 0.4μL of each primer at concentrations of 0.2 μM, 0.2μL Mg buffer at a concentration of 0.5 mM to top-up the one present in the BIONEER, and 6.5–7.5 μL ddH_2_O.PCR amplification was carried out with the following conditions: initial denaturation at 94°C for 3 min, followed by 30 cycles of denaturation at 94°C for 30s, annealing at 55°C—65°C (depending on the particular primer) for 30s to 1min and extension at 72°C for 2min; and a final extension at 72°C for 15 minutes. Annealing temperatures of each primer were determined by primer digital software (primerdigital.com). PCR products of contrasting florescent labels were pooled based on dye color and band intensities to have uniform signal strength. The pooled PCR amplicons were denatured with Hi-Di formimide at 95°C for 3min and then separated by capillary electrophoresis using an ABI3730 DNA genetic analyzer (Applied Biosystems, Foster City, CA) using GeneScan- 500 Internal LIZ and 1200 Internal LIZ Size Standards based on size of amplicons. The fragment analysis data from ABI3730 system were analyzed and allele sizes scored with GENEMAPPER V 4.1software (Applied Biosystems).

### 2.3 Genetic diversity analysis

The genotypic data were subjected to various within and among groups genetic diversity measures. Basic statistics including: major allele frequency, total number of alleles, gene diversity and polymorphism information content (PIC) using PowerMarker genetic analysis package (version 3.25) [35]; and number of private alleles/accession-specific alleles, number of effective alleles, observed heterozygosity, expected heterozygosity and shannon's Information Index using GenAlEx version 6.5 [36]; were calculated to measure allelic diversity. Furthermore, abundance of alleles (number of rare alleles, common alleles and abundant alleles) and partitioning of total genetic variation into within and among pre-grouped populations through molecular analysis of variance (AMOVA)were computed using GenAlEx version 6.5 [36].

### 2.4 Genetic relationships analysis

Genetic distances between each pair of accessions and between pre-grouped populations were measured based on both shared allele frequencies and Nei's genetic distance [37]using PowerMarker [35]. Genetic distance matrices for each locus were summed across loci assuming statistical independence. Pair-wise genetic frequency based dissimilarity or distance matrix between individuals was calculated according to Nei et al.[37] as implemented in PowerMarker. The resulting dissimilarity matrix was subjected to tree construction using the un-weighted pair group method analysis (UPGMA) using the same software.

### 2.5 Construction of core collections

Core collections are a group of accessions from an existing germplasm collection that is chosen to represent the genetic spectrum of the entire collection [13]. Core collections formed for the purposes of maximizing the representativeness of genetic diversity as suggested by Marita et al. [38] ensure that all accessions in the entire collection are maximally represented, and thus provide the best option for obtaining a single ‘‘multipurpose” or generalist core collection compared to any other type [39].To construct the genetic core collections we used allelic data to calculate the dissimilarity matrix as implemented in DARwin v 6.0.10 software[40] following random sampling method. Individual members of the core collection were selected based on the number of alleles that they contribute for and based on standard deviation from the mean. Landrace accessions that when removed do not reduce the total number of alleles of the entire collection were not included in the core collection. The size of the core collection and efficiency of the strategy was assessed by comparing the total number of alleles captured for each run using the same software. The size of the core collection was expressed as a proportion of the number of individuals selected for the core collection to the number of individuals in the entire collection.

### 2.6 Population differentiation and structure analysis

To study population differentiation, pair-wise Fst among all pairs of populations were computed [41]. The model-based software Structure 2.3.4[42, 43] was used to infer the population structure for the sampled landrace set using a burn-in of 10,000, a run length of 100,000, and a model allowing for admixture and correlated allele frequencies. At least five runs of Structure were conducted by setting the number of populations (K) from 1 to 20. The model choice criterion to detect the most probable value of K was, both the LnP(D) value for each given K and ΔK, an *ad hoc* quantity related to the second-order change of the log probability of data with respect to the number of clusters inferred by Structure[44]. Once the best K was found, the analysis was re-run in the same software using a burn-in of 10,000, a run length of 500,000 with the same aforementioned model. CLUMPAK: "a program for identifying clustering modes and packaging population structure inferences across K" (CLUMPAK server) was used[45].

## Result

### 3.1 Genetic diversity

Two hundred and twelve Ethiopian white lupin landrace accessions and the two out-group genotypes were amplified using 16 SSR markers, which successfully amplified across all accessions. However, one marker (Lup146) produced very large fragment size, which could not be sized with the available largest GeneScan- 1200 LIZ Size Standard at the BecA-ILRI Hub and hence was eliminated from genotyping. Moreover, the remaining 15 SSR markers were checked for medium to high polymorphism before embarking on further analysis. The 15 SSR markers revealed 108 alleles, of which 98 alleles were from the 212 white lupin landrace accessions and 10 alleles from out-group genotypes.SSR locus diversity data are summarized and provided in Table 1. The number of alleles (NA) per locus varied among the markers, ranging from 3 (LSSR55 &GLNA) to 12 (Lup197& Lup257), with an average of 6.53 alleles. The major allele frequency (MAF) per locus ranged from 0.48 (CHS9) to 0.95 (Lup125), with an average of 0.80 per marker. The total microsatellite genotypes were 138, ranging from 4 (LSSR55, Lup125, GLNA & DSI) to 23 (Lup257). Among the 15 SSR markers, the overall Polymorphism information content (PIC) values ranged from 0.10 (Lup125) to 0.63 (Lup197), with an average of 0.28. The observed heterozygosity (Ho) values ranged from zero (Lup125 and DSI) to 0.96 (CHS9), with an average of 0.18, and the expected heterozygosity (He) values ranged from 0.1 (Lup125) to 0.67 (Lup197), with an average of 0.31 (Table 1).

**Table 1:** Statistics of genetic diversity across 15 SSR loci in the 212 white lupin landrace accessions and the out-group genotypes.

Marker	MAF	NG	NA	NPA	Ne	I	He/GD	Ho	PIC
LSSR55	0.91	4	3	1	1.26	0.32	0.16	0.15	0.14
PT1-1	0.87	10	6	2	1.43	0.50	0.24	0.19	0.22
LSSR26a	0.80	5	4	2	1.50	0.47	0.33	0.32	0.28
PT1-2	0.89	9	5	1	1.35	0.41	0.20	0.15	0.19
LSSR14	0.91	6	5	0	1.21	0.36	0.17	0.17	0.16
La1-EST01	0.90	10	6	1	1.36	0.52	0.19	0.11	0.19
Lup125	0.95	4	4	2	1.16	0.26	0.10	0.00	0.10
AnMtS13	0.85	6	5	1	1.54	0.56	0.26	0.11	0.25
GLNA	0.89	4	3	0	1.41	0.47	0.21	0.08	0.20
DSI	0.91	4	4	1	1.21	0.32	0.17	0.00	0.16
CHS9	0.48	11	8	1	2.29	0.93	0.56	0.96	0.46
LSSR9a	0.75	13	11	6	1.18	0.80	0.42	0.12	0.40
Lup257	0.72	23	12	1	1.98	1.03	0.46	0.09	0.44
LSSR9b	0.73	13	10	5	1.85	0.88	0.45	0.15	0.42
Lup197	0.51	16	12	4	2.91	1.31	0.67	0.09	0.63
Total		138	98	28					
Mean	0.80	9.20	6.53	1.9	1.58	0.61	0.31	0.18	0.28

MAF, major allele frequency; NG, number of genotypes; NA, number of alleles; NPA, number of private allele; Ne, number of effective alleles; I, Shannon's Information Index; He, expected heterozygosity; Ho, observed heterozygosity; PIC, polymorphic information content.

Twenty eight landraces harbored one or more private alleles (Table 2) from the total of 28 private alleles identified in the 212 white lupin accessions(Table 1). Thirteen of the 15 SSR loci contained one or more of the private alleles identified (Table 1). Private alleles are the number of unique alleles in a population, which is a simple measure of genetic distinctiveness [46].The RA (rare allele) ranged from 1 (GLNA) to 10 (Lup257 and Lup197) with a total of 77,with each allele having a frequency less than 5%. It accounted for 78.6% of the total allele detected (Table 2). Eight of the SSR loci (LSSR55, PT1-1, PT1-2, LSSR26a, La1-EST01, Lup125, CHS9 and Lup197) did not contain common alleles, whereas two loci (LSSR9a and LSSR9b) possessed two common alleles each, with a frequency of 5–50%.

**Table 2:** Detail of allele types identified and landraces containing private alleles as revealed by 15 SSR loci among the 212 white lupin landrace accessions.

Marker	R_A_	C_A_	A_A_	Total	Accessions containing one or more private alleles
LSSR55	2	0	1	3	Aus14, Aus19, Aus17, Aus9, Acc30, Acc10, Acc9, Acc158, Acc12, Acc24, Acc118, Acc107, Acc122, Acc164, Acc110, Acc114, Acc131, Acc44, Acc39, Acc143, Acc41, Acc70, Acc102, Acc100, Acc101, Acc177, Acc170, Acc96
PT1-1	6	0	0	6	
LSSR26a	4	0	0	4	
PT1-2	5	0	0	5	
LSSR14	3	1	1	5	
La1-EST01	5	0	1	6	
Lup125	3	0	1	4	
AnMtS13	3	1	1	5	
GLNA	1	1	1	3	
DSI	2	1	1	4	
CHS9	7	0	1	8	
LSSR9a	9	2	0	11	
Lup257	10	1	1	12	
LSSR9b	7	2	1	10	
Lup197	10	0	2	12	
Total	77	9	12	98	28

R_A_, number of rare (<5%) alleles; C_A_, number of common (5–50%) alleles; A_A_, number of abundant (>50%) alleles.

The genetic diversity indices of populations are summarized and provided in Table 3. Among the Ethiopian white lupin pre-defined populations, the number of different alleles and number of private alleles were highest in the West Gojam population and lowest in the Australian donations and Gondar populations. However, Australian donations showed the highest number of effective alleles, expected heterozygosity and Shannon Information Index (Table 3).

**Table 3:** Summary of the population diversity indices averaged over 15 loci.

Population	Genetic diversity parameters				
	Na	Ne	NPA	He	I
West Gojam	4.73	1.45	0.67	0.24	0.51
East Gojam	3.80	1.54	0.40	0.27	0.56
Awi	4.40	1.66	0.40	0.34	0.69
Gondar	3.40	1.41	0.13	0.22	0.45
Australia*	3.60	2.04	0.13	0.48	0.85
Out-group	1.27	1.24	0.67	0.21	0.30
Average	3.53	1.56	0.40	0.29	0.56

Australia*, Australia collections & donations; NA, number of alleles; Ne, number of effective alleles; NPA, number of private allele; He, expected heterozygosity; I, Shannon's Information Index.

The analysis of molecular variance showed that 92% of allelic diversity was attributed to individual accessions within populations while only 8% was distributed among populations (Table 4). No significant variation for molecular diversity was observed among populations based on areas of collection depicting shared alleles among them. However, accessions collected by Australians followed by those from Awi had fairly higher level of gene diversity and Shannon's diversity index.

**Table 4:** Analysis of molecular variance (AMOVA) in Ethiopian white lupin landrace populations.

Source of variations	Degree of freedom	Sum square	Mean square	Estimated variances	Proportion of explained variance	Statistics	value	P value
Among Populations	4	132.41	33.1	0.66	8%			
Within Populations	207	1491.63	7.21	7.21	92%	PhiPT	0.084	0.001
Total	211	1624.03		7.87	100%			

### 3.2 Genetic relationships

Frequency based genetic distances between all pair-wise combinations among the 212 white lupin accessions and the out-group genotypes were calculated as per Nei et al. [37] distance method PowerMarker software. Pair wise genetic distance ranged from 0 (accessions are more similar) to 1.0 (between some Ethiopian accessions with the out-group genotypes) indicating the presence of genetic diversity among accessions.

**Fig 2:**
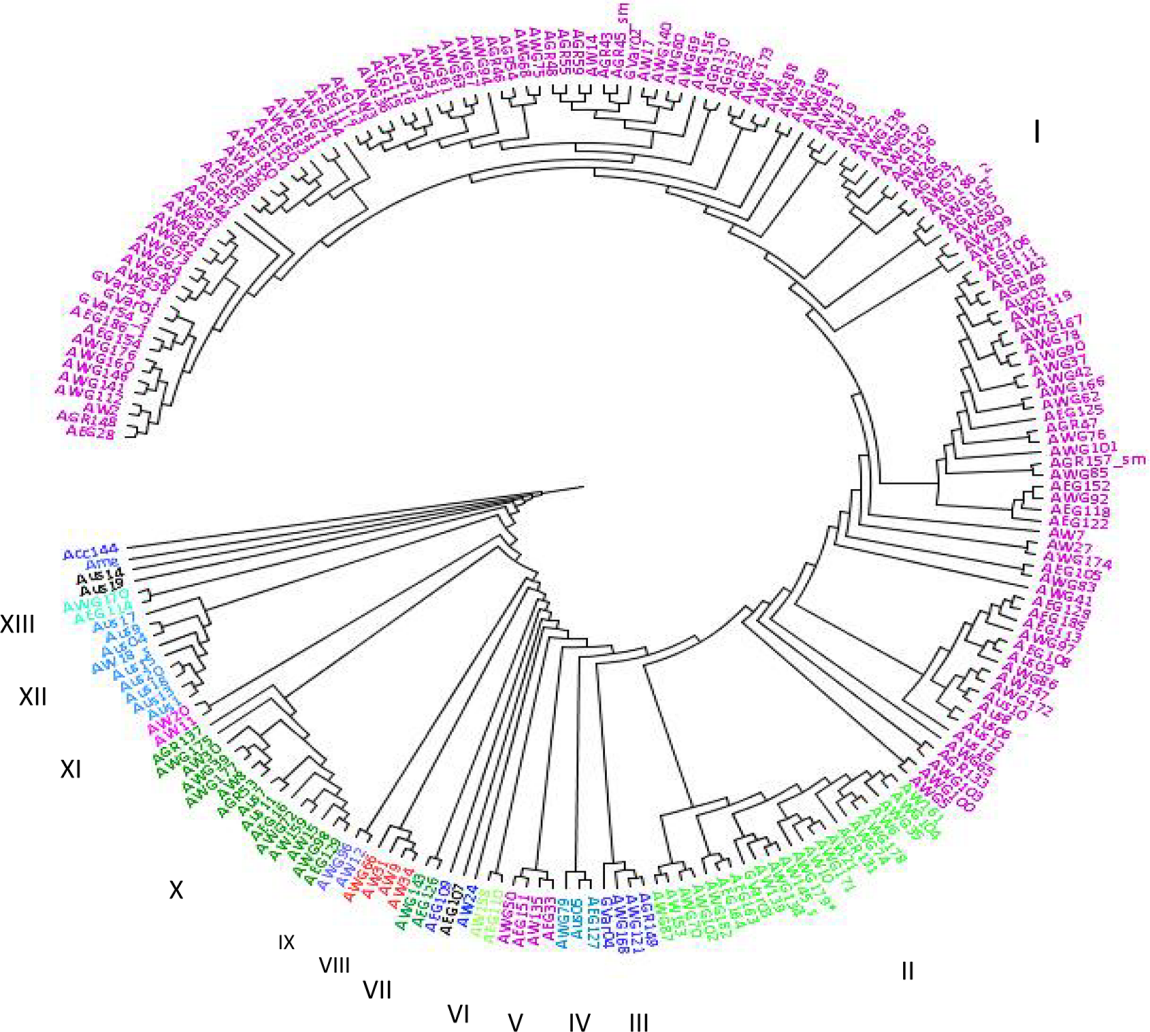
UPGMA dendrogram showing the genetic relationships among the 212 Ethiopian white lupin landrace accessions and the out-group genotypes.

The resulting distance matrix was used to construct an UPGMA tree. The generated dendrogram revealed a complex accession distribution pattern with no clear grouping based on geographic origin. At 70% similarity level, the UPGMA dendrogram resulted in the formation of 13 clusters comprised of 2 to 136 landraces, with the out-group genotypes and five landraces remaining distinct and ungrouped (Fig 2; Table 5). The largest cluster, cluster I is constituted of 136 landrace accessions with the following proportions from the different collection areas in the country: West Gojam (63); Gondar (26); East Gojam (23); Awi (17); and Australia donated (7). The second largest cluster comprised 22 landraces the majorly of which are from West Gojam (11) and Awi (7), and two each from East Gojam and Gondar. The third largest cluster is cluster X that harbored 15 landrace accessions from all the collection areas. The dendrogram did not show any clear divisions among the white lupin accessions based on their geographical locations except that West Gojam and Gondar which are neighboring areas shared most accessions and clustered together as clearly shown in cluster I.

**Table 5:** Clustering patterns of Ethiopian white lupin landraces from different origins over the clusters.

Areas of collection/Origin	No of accessions	Number of landraces in each cluster													
		I	II	III	IV	V	VI	VII	VIII	IX	X	XI	XII	XIII	Singletons
West Gojam	87	63	11	2	1	1	-	1	1	1	5	-	-	1	-
East Gojam	35	23	2	-	1	2	1	1	-	-	2	-	-	1	2
Awi	38	17	7	-	-	1	1	-	3	1	4	2	1	-	1
Gondar	32	26	2	2	-	-	-	-	-	-	2	-	-	-	-
Australia*	20	7	-	-	1	-	-	-	-	-	2	-	8	-	2
Out-group	2	-	-	-	-	-	-	-	-	-	-	-	-	-	2
Total	214	136	22	4	3	4	2	2	4	2	15	2	9	2	7

Australia*, Australia collections & donations; -, Nil

**Fig 3:**
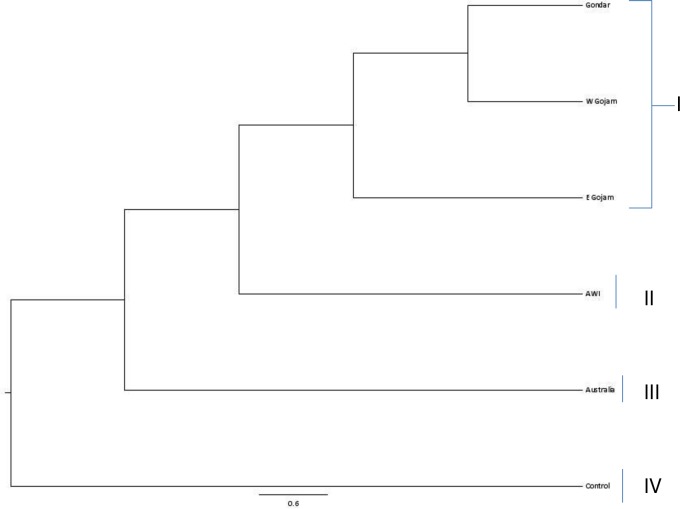
UPGMA dendrogram showing the genetic relationships among the Ethiopian white lupin landrace populations collection areas.

For the different areas of collection, genetic distances between populations from different parts of Ethiopia and the out-group were greater than any other combinations of paired population. Gondar population is the most similar to population of West Gojam (0.027), whereas population collected by Australians showed the greatest genetic distance with population of Gondar (0.147). An UPGMA tree of the six collection areas including the out-group was constructed based on Nei’s genetic distances and resulted in the formation of three groups with regard to areas of collection or origin (Fig 3). The first group contained Gondar, West Gojam and East Gojam. Awi and population collected by Australians were grouped into the second and third groups, respectively. The out-group isolated apart and clustered in a separate and different group. The dendrogram revealed patterns of genetic relationship among proximity areas of collection.

### 3.3 Construction of core collection

Genetic core collections were constructed among Ethiopian white lupin landrace accessions based on 15 polymorphic SSR markers. The study revealed that thirty four accessions (core G-34) were sufficient to retain 100% of SSR diversity, i.e. captured all the 98 alleles which were detected from the 212 Ethiopian white lupin landraces. These accessions (core G-34) represent 16% of the whole 212Ethiopian white lupin accessions considered in this study. Detailed descriptions of the thirty four white lupin landraces which is proposed to constituent national core collection for Ethiopian white lupin landrace accessions are provided in Table 6. This result shows that only a small number of accessions are needed to retain the whole allelic diversity of Ethiopian white lupin landrace populations.

**Table 6:** Description of Ethiopian white lupin landrace accessions proposed to constituent national core collection for Ethiopian white lupin accessions.

Acc No	IBC code	Zone	District	Altitude	Acc No	IBC code	Zone	District	Altitude
AEG122	105015	EG	Machakel		AWG102	238999	WG	Mecha	2050
AEG129	242260	EG	Machakel	2400	AWG103	239001	WG	Mecha	2050
AEG164	216015	EG	Machakel	2280	AWG162	239031	WG	Achefer	2010
AGR131	239012	NG	G zuria	1930	AWG169	239036	WG	Achefer	2000
AGR46	242313	SG	Dera	1960	AWG170	239037	WG	Achefer	2000
Aus14	Australia collections and donations				AWG177	239048	WG	Bure W	2600
Aus17					AWG179	239050	WG	Bure W	2300
Aus7					AWG39	239011	BD Sp	Bahir Dar	2090
Aus9					AWG41	239022	BD Sp	Bahir Dar	1930
AW15	242276	AWI	Banja	2590	AWG66	242297	WG	Achefer	2010
AW158	242289	AWI	Fagta	2375	AWG74	239030	WG	Achefer	2010
AW18_s		AWI			AWG79	242310	WG	Bd Z	1880
AW21	242292	AWI	Dangila	2060	AWG83	239046	WG	Bure w	2520
AW24	236617	AWI	Dangila	2040	AWG92	242270	WG	Dembecha	2050
AW30	242287	AWI	Fagta	2550	AWG96	242305	WG	Mecha	2000
AW9	242283	AWI	Banja	2160	GVar03	Variants identified while phenotyping the IBC collections			
AWG101	238997	WG	Mecha	2060	GVar04				

Acc No, Accessions number given in the study; EBI, Ethiopian Biodiversity Institute; WG, West Gojam; EG, East Gojam; Bd Z, Bahir Dar zuria; Bure W, Bure Womberema; BD SP, Bahir Dar Special; NG, North Gondar; SG, South Gondar; G zuria, Gondar Zuria; Fagta, Fagta Lekoma.

### 3.4 Population differentiation and structure

Different population genetics parameters including genetic differentiation (Fst), Nei's unbiased genetic distance and Nei's genetic identity were analyzed using GenAlEx 6.5 [36]. *F*st values among pairs of populations ranged from 0.008 (between West Gojam and Gondar) to 0.472 (between Gondar and the out-group) with an overall average of 0.179 (Table 7). Population differentiation was higher between the out-group and any of the five populations than any pair combination of the five populations. Nei’s unbiased genetic distance was high between the out-group and any of the five populations than any pair combination of the five populations. Among the five landrace populations, relatively high genetic distance was exhibited between Gondar and population collected by Australians. Genetic identity was very high among the five populations (0.858–0.999); and very low between the out-group and any of the five populations (0.345–0.358).

**Table 7:** Pair-wise genetic differentiation, unbiased Nei’s genetic distance and identity among populations based on areas of collection in Ethiopian white lupin landraces.

Populations	GI						GD					
	Au	AWI	OG	EG	GR	WG	Au	AWI	OG	EG	GR	WG
Au (N = 20)		0.89	0.35	0.89	0.86	0.88		0.11	1.04	0.11	0.15	0.13
AWI (N = 38)	0.08		0.36	0.98	0.99	1.00			1.03	0.02	0.01	0.01
Out-group (N = 2)	0.37	0.42		0.35	0.35	0.35				1.05	1.06	1.07
E Gojam (N = 35)	0.09	0.02	0.45		0.99	1.00					0.01	0.01
Gondar (N = 32)	0.13	0.02	0.47	0.02		1.00						0.00
W Gojam (N = 87)	0.11	0.01	0.47	0.01	0.01							
	Fst											

Au, Australia collections &donations; OG, Out-group; EG, East Gojam, GR, Gondar; WG, West Gojam. Nei’s unbiased genetic identity (GI) and unbiased Nei’s genetic distance (GD) in upper diagonals in right and left of the table; Genetic differentiation (Fst) in lower diagonal in left of the table.

**Fig 4:**
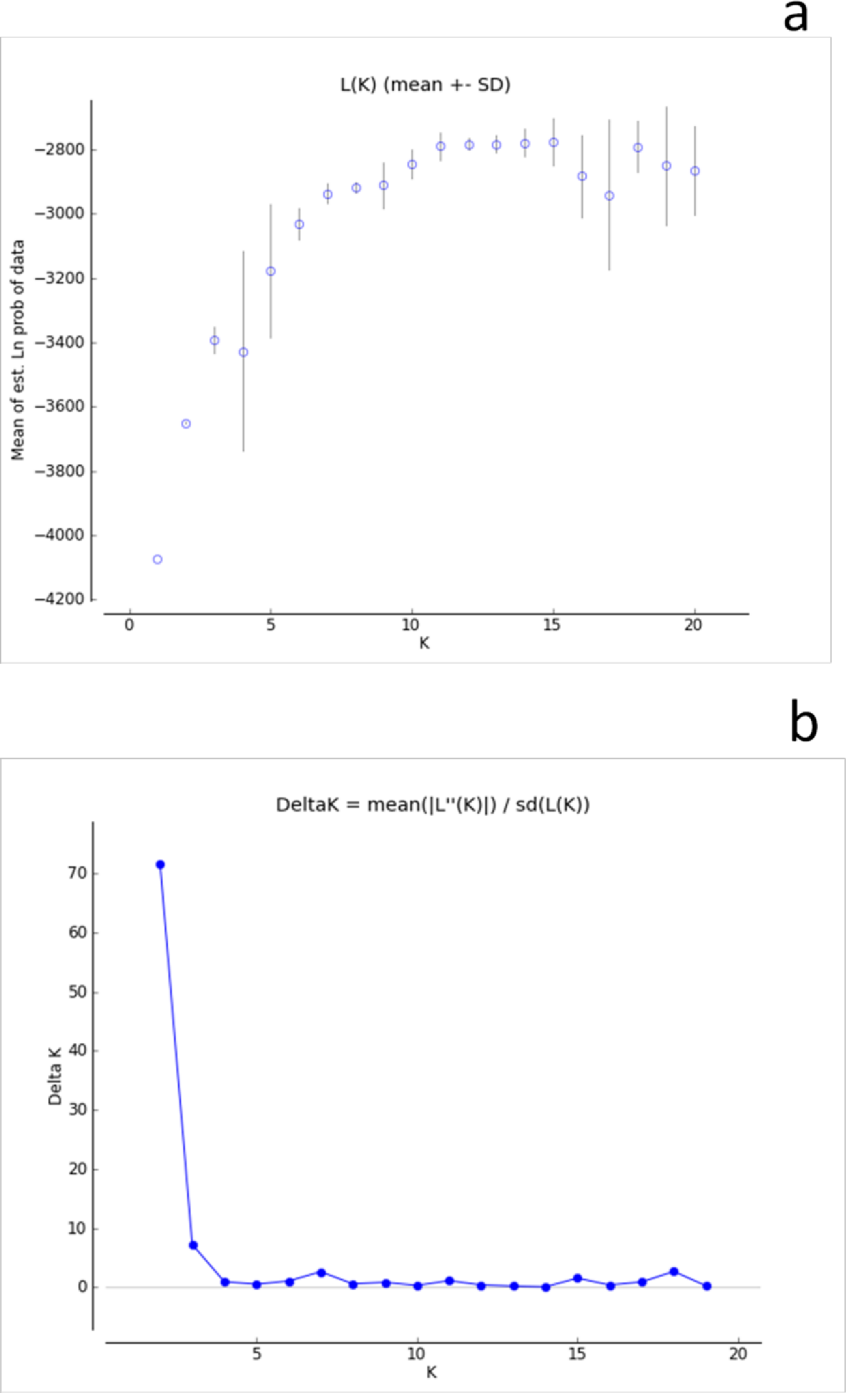
The true value of K inferred from first and second order statistics:(a)Log-likelihood plots; (b). ΔK from structure analysis of 214 lupin accessions.

**Fig 5:**
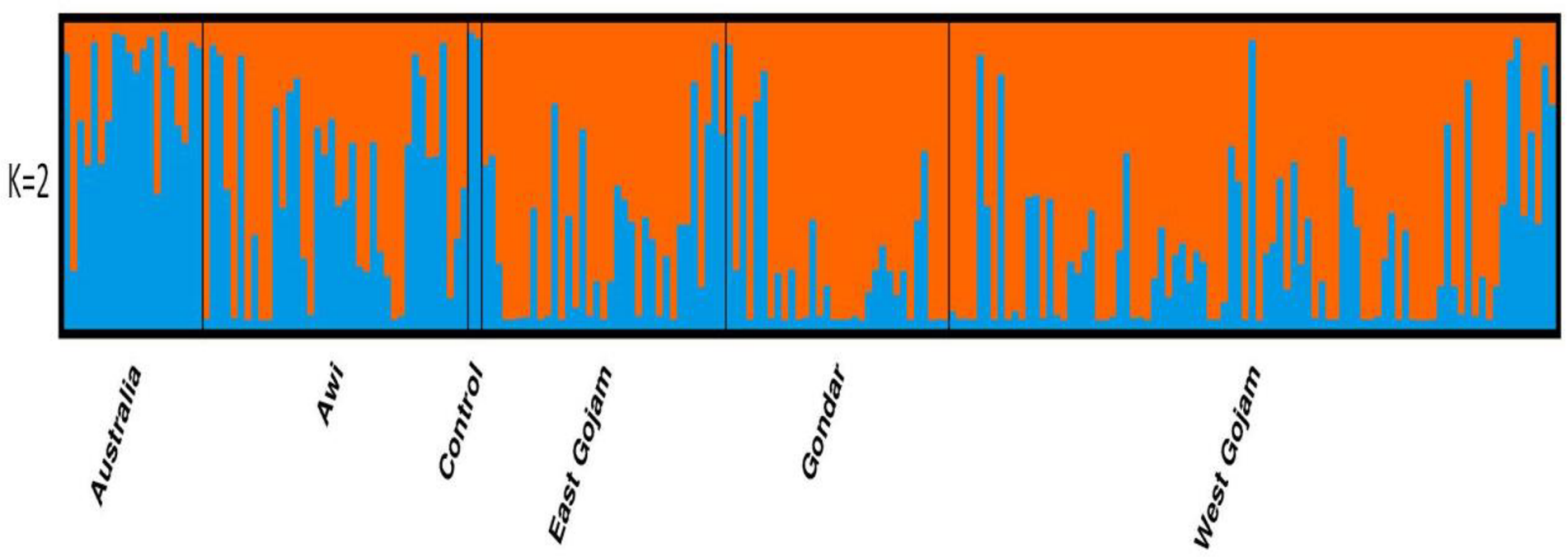
Model based population structure of 212 Ethiopian white lupin landraces based on 15 SSR panels as obtained from Structure2.3.4 software.

A total of 15 SSR markers were used to analyse the population structure of the panel of 212white lupin landrace accessions and out-group genotypes using a model-based approach in Structure, giving 1 to 20 possible clusters (K). The results were then permuted for each K value using the Structure software; and the results were collected through structure harvester [47]. The LnP(D) value for each given K increased with the increase in K, but as there was no clear change in the LnP(D) value, the probable K value could not be inferred (Fig 4A). However, on applying the second-order statistics(ΔK) developed by Evanno et al. [44], a sharp peak in ΔK at K = 2 was observed, suggesting the presence of two major populations (Fig 4B; S3 Table). The analysis for K = 2 populations showed individual landrace accessions from the five collection areas distributed between the two populations (Fig 5; Table 8).However, significant proportion of the populations, 74% from West Gojam, 78% Gondar, 70% East Gojam and 56% Awi grouped together in population one with significant admixtures; whereas the majority (more than 75%) of those collected by Australians and 44% of Awi grouped to a second population (Table 8). Generally, this model based grouping was somehow congruent with UPGMA.

**Table 8:** Proportion of membership of each predefined population, in each of the clusters obtained at the best k, i.e., k = 2.

Pre-defined Populations	Number of accessions	Cluster I		Cluster II	
		Proportion	number	Proportion	number
Australia*	20	22.90	5	77.10	15
Awi	38	55.90	21	44.10	17
Out-group	2	4.10	0	95.90	2
East Gojam	35	70.40	25	29.60	10
Gondar	32	78.60	25	21.40	7
West Gojam	87	74.10	64	25.90	23
Total accessions	214		140		74
Average distance**		0.10		0.58	

Australia*, Australia collections & donations; Average distance**, Average distance (expected heterozygosity) between individuals in the same cluster.

## Discussion

### 4.1 Genetic diversity and SSR allelic distribution

In this study, SSR polymorphism was high as revealed by the high allelic richness per locus, which varied widely among the markers from 3 to 12 alleles (average 6.5 alleles).Relatively moderate to high PIC, Ho and He values were also observed in most markers as well as high number of private alleles and high proportion of rare alleles, indicating a high level of genetic diversity in the Ethiopian lupin landraces studied. Our results are supported by previous studies based on morphological and agronomic traits, ISSR, AFLP, SSR and DArT marker systems analyses, which reported high genetic diversity in white lupin landraces in Ethiopia[23, 31,32]and in other parts of the world including Egypt [48], Morocco [49]and Spain [14, 50]. Similarly, Gwag et al. [51] reported high frequency of rare alleles (34 alleles; 51.5%) among mung bean accessions, and also indicate that these private alleles contributed greatly to the overall genetic diversity of the collection in other crops [52, 53]. Hence, it is important to include rare alleles to maximize the genetic variation in gene bank collections and to utilize them in a breeding program [53]. The high orientation of allelic diversity resided on individuals within populations than among populations in this study is in good agreement with previous studies on white lupin using ISSR markers though their sample size was too small (only 39) [32] and on common bean using SSR markers [54]. However, relatively high molecular variance (59%) among populations was reported on chickpea [55] from diversified populations including wild relatives. The relatively higher variance obtained among populations could be attributed to the presence of wild relatives included in the study. Some reports indicate that the level of polymorphism depends on the type of germplasm [56], floral biology, marker used [57, 58], primers selected [58, 59] and the sampling strategy [59].

### 4.2 Genetic relationships and patterns of clustering

The dendrogram based on UPGMA revealed two major clades with a complex accession distribution pattern. Nevertheless, at about 70% similarity, the UPGMA dendrogram revealed 13 clusters comprised of 2 to 136 landraces while the out-group genotypes and five landraces remained distinct and ungrouped. Similar relationship and clustering pattern were reported for Ethiopian white lupin landraces based on agronomical and phenological traits [31]. Raman et al. [23] also found comparable clustering patterns of white lupin accessions originated from different countries including Ethiopia. Sixty four percent of the landraces (136) were grouped in a single cluster (cluster I) and this cluster was constituted by 81% from Gondar, 72% of West Gojam and 65% of East Gojam. This is not surprising since these are geographically bordering areas. This result was further confirmed with low population differentiation among the four major white lupin collection areas in Ethiopia; namely, West Gojam, Awi, East Gojam and Gondar. Moreover, this result is clearly reflected on the model based structure analysis findings showing significant admixtures of gene pool across populations. Among the five landrace populations, relatively high genetic distance exhibited between population of Gondar and population collected by Australians. This finding may indicate seed exchange, and/or trade between farmers, leading to gene flow across boundaries within those areas[60].

Nevertheless, the dendrogram did not indicate any clear divisions among the white lupin accessions based on their geographical locations. A supportive result is documented by Sbabou *et al*. [49]who found that white lupin local accessions in Morocco clustered regardless of their geographic origin. Distribution of accessions of similar origin into different clusters might indicate the existence of accession diversity within the populations of origin. The distributions of accessions from West Gojam, Awi and East Gojam, over different clusters, were high covering 10, 9, and 9 clusters, respectively. Moreover, three out of the five singletons were from these areas. This might indicate that accessions from these areas are more diverse than others. The distribution and pattern of accessions, over clusters from these three major geographic origins, would suggest future collections of local accessions in those geographic regions. This result is consistently in agreement with the agronomic and phenologic characterization of subset of these landraces [31]; and it is further supported by Raman et al.[23] who showed that Ethiopian accessions formed a very distinct and separate grouping/gene pool than others. On the other hand, accessions from non-bordering regions grouped together in particular clusters as similarly observed through a study on local field pea and faba bean accessions in Ethiopia, and reported by Keneni*et al*. [61]. One possible reason could be that the landraces might have been introduced from a similar origin.

Cluster analysis of populations based on areas of collection showed that population grouping did not follow strictly geographical proximity. Therefore, we evaluated them to understand whether alternative clustering methods such as model based clustering would resolve similar patterning. From the dendrogram analysis, it was observed that there were two major populations corresponding to the major collection areas on one hand and populations collected by Australians on the other. By the Structure analysis, these two major populations were resolved but with significant admixtures. The dendrogram analysis also provided some evidence for gene flow between the subpopulations. Generally, there was good correspondence between the population pattern observed in the dendrogram and the population structure identified using Structure.

### 4.3 Construction of core collections

The sampling percentage of a core collection has long been under debate. Brown [62] suggested a sampling percentage of 5%–10%. Yonezawa et al. [63] thought 20%–30% of the sampling percentage was needed to well conserve the genetic diversity of the whole germplasm collection. The present study resulted in 16% proportion of core collections to the base collections. Slightly higher sampling percentage than the present study was reported by previous researchers, for example, 22% in dry bean [64]and 27% in faba bean [65].In general, most core collection sizes are 10%–30% of the initial collection [66–68]. However, a perfect ratio or fixed size for all core collections does not exist, and different plant or different constructing goals need different sampling percentage[69].Our results demonstrated the great potential of using molecular data to construct a core collection and thus improve the management and utilization of the Ethiopian white lupin landrace germplasm collections.Hu et al.[70] showed that core collection based on genotypic values retained larger genetic variability and had superior representatives than those based on phenotypic values. Nevertheless, because we used a relatively small number of genic and genomic SSR markers for the genetic analysis, the data presented here should not be used alone when deciding on which accessions from the germplasm collection should be discarded or maintained. Additional molecular markers, including more SSRs and single nucleotide polymorphisms (SNPs), should be used to provide better coverage of the genome. Moreover, this information should be coupled with agronomic, morphological and structural data to make a final decision on the accessions to be maintained.

Finally, although molecular marker technologies including SSRs have been widely applied for germplasm characterization and diversity studies, there are limitations on their application mainly because of their limited abundance in the genome. Next-generation sequencing (NGS) approaches which enable genome-wide marker analysis coupled with high-throughput genotyping platforms holds promise to significantly increase our understanding of the genomic information that could be considered in designing strategies to crop improvement. Furthermore, it has been possible to develop diverse next-generation-based reduced representation protocols, which could be optimized to any crop species with or without a reference genome sequence [71]. Such protocols are important for the simultaneous discovery and generation of massive, genome-wide SNPs, which have a wide array of applications, including diversity studies, population genetics and QTL analysis[72]. Genotyping by sequencing (GBS) is among the most widely used complexity reduction multiplex genotyping strategies[73], and has been successfully used to discover SNP markers associated with important traits in many crops including anthracnose resistance in lupin [74].

## Conclusion

The study showed that genotyping combined with clustering and population structure analysis is a powerful method for characterizing germplasm. It was also able to access and evaluate some polymorphic SSR loci which can be effectively used in future genetic analysis studies and molecular breeding programs. Further, this study confirmed that genetic diversity exists in the Ethiopian white lupin landrace accessions with several rare alleles. Nevertheless, the result also claims much redundancy in the landrace collections as more than 64% of them clustered together and only 34 accessions are sufficient to retain 100% SSR diversity i.e. captured all the 98 alleles which appeared to be present in the whole accessions considered for this study. The extent and pattern of the existing genetic diversity does not strictly follow the geographic origin or areas of collection. Generally, the results suggest that future breeding programs could exploit the available genetic diversity harbored in the Ethiopian white lupin landraces and to design and implement appropriate conservation strategies including core collection establishment and future national collection missions.

## Supporting information

S1 Table Description of the Ethiopian white lupin landrace accessions and the out-groups used for the study. (DOCX)

S2 Table List of SSR markers used for the study. (DOCX)

S3 Table The Evanno table output. (DOCX)
