# Supplementary material for: Molecular genetic diversity and population structure of Ethiopian white lupin landraces: Implications for breeding and conservation
Source: PLoS One. 2017 Nov 30;12(11):e0188696. doi: 10.1371/journal.pone.0188696 (PMC5708786; doi:10.1371/journal.pone.0188696)
Supplement: S1 Table — (DOCX) [file pone.0188696.s001.docx]

S1 Table. Description of the Ethiopian white lupin landrace accessions and the out-group genotypes used for the study.

| Acc no | EBI code | Zone | District | Altitude | ACC no | EBI code | Zone | District | Altitude |
| --- | --- | --- | --- | --- | --- | --- | --- | --- | --- |
| Acc1 | 242279 | Awi | Ankesha | 2310 | Acc37 | 238993 | BD Sp | Bahir Dar | 1990 |
| Acc2 | 242280 | Awi | Ankesha | 2185 | Acc38 | 238994 | BD Sp | Bahir Dar | 2020 |
| Acc3 | 242281 | Awi | Ankesha | 2310 | Acc39 | 239011 | BD Sp | Bahir Dar | 2090 |
| Acc4 | 242282 | Awi | Ankesha | 2410 | Acc40 | 239020 | BD Sp | Bahir Dar | 1940 |
| Acc5 | 242266 | WG | Dembecha | 2110 | Acc41 | 239022 | BD Sp | Bahir Dar | 1930 |
| Acc6 | 239044 | Awi | Banja | 2600 | Acc42 | 239023 | BD Sp | Bahir Dar | 1930 |
| Acc7 | 242277 | Awi | Banja | 2560 | Acc43 | 228519 | SG | Dera |  |
| Acc8 | 242278 | Awi | Banja | 2560 | Acc44 | 242311 | SG | Dera | 1860 |
| Acc9 | 242283 | Awi | Banja | 2160 | Acc45 | 242312 | SG | Dera | 1960 |
| Acc10 | 242284 | Awi | Banja | 1960 | Acc46 | 242313 | SG | Dera | 1960 |
| Acc11 | 236619 | Awi | Banja | 2570 | Acc47 | 242314 | SG | Dera | 2160 |
| Acc12 | 239045 | Awi | Banja | 2600 | Acc48 | 242315 | SG | Dera | 2380 |
| Acc13 | 242273 | Awi | Banja | 2490 | Acc49 | 242316 | SG | Dera | 2460 |
| Acc14 | 242274 | Awi | Banja | 2450 | Acc50 | 242268 | WG | Dembecha | 2010 |
| Acc15 | 242276 | Awi | Banja | 2590 | Acc51 | 239018 | WG | BD Z | 1950 |
| Acc16 | 105018 |  | Unknown |  | Acc52 | 242319 | SG | Dera | 2510 |
| Acc17 | 105005 | Awi | Dangila | 1940 | Acc53 | 105002 | SG | Este | 2420 |
| Acc18 | 228520 | Awi | Dangila |  | Acc54 | 226034 | SG | Este | 2560 |
| Acc19 | 242290 | Awi | Dangila | 2240 | Acc55 | 242321 | SG | Este | 2630 |
| Acc20 | 242291 | Awi | Dangila | 2160 | Acc56 | 242219 | SG | Farta | 2280 |
| Acc21 | 242292 | Awi | Dangila | 2060 | Acc57 | 242322 | SG | Farta | 2850 |
| Acc22 | 242293 | Awi | Dangila | 2100 | Acc58 | 242323 | SG | Farta | 2760 |
| Acc23 | 242294 | Awi | Dangila | 2060 | Acc59 | 212754 | SG | Fogera | 1950 |
| Acc24 | 236617 | Awi | Dangila | 2040 | Acc60 | 239008 | WG | Achefer | 2070 |
| Acc25 | 239003 | Awi | Dangila | 2190 | Acc61 | 239029 | WG | Achefer | 2030 |
| Acc26 | 239004 | Awi | Dangila | 2220 | Acc62 | 239033 | WG | Achefer | 2000 |
| Acc27 | 239005 | Awi | Dangila | 2360 | Acc63 | 239038 | WG | Achefer | 2150 |
| Acc28 | 242253 | EG | Machakel | 2140 | Acc64 | 242295 | WG | Achefer | 2050 |
| Acc29 | 239007 | Awi | Dangila | 2190 | Acc65 | 242296 | WG | Achefer | 1975 |
| Acc30 | 242287 | Awi | Fagta | 2550 | Acc66 | 242297 | WG | Achefer | 2010 |
| Acc31 | 242288 | Awi | Fagta | 2425 | Acc67 | 242298 | WG | Achefer | 1990 |
| Acc32 | 239017 | SG | Dera | 2130 | Acc68 | 242299 | WG | Achefer | 2000 |
| Acc33 | 242254 | EG | Machakel | 2150 | Acc69 | 242300 | WG | Achefer | 2060 |
| Acc34 | 242286 | Awi | Guangua | 1740 | Acc70 | 242301 | WG | Achefer | 2090 |
| Acc35 | 105003 | BD Sp | Bahir Dar | 1790 | Acc71 | 242302 | WG | Achefer | 2000 |
| Acc36 | 239021 | BD Sp | Bahir Dar | 1940 | Acc72 | 239009 | WG | Achefer | 2000 |

Acc no, Accession number; EBI, Ethiopian Biodiversity Institute; WG, West Gojam; EG, East Gojam; BD SP, Bahir Dar Special; SG, South Gondar; Fagta, Fagta Lekoma.

S1 Table. (Continued)

| Acc no | EBI code | Zone | District | Altitude | Acc no | EBI code | Zone | District | Altitude |
| --- | --- | --- | --- | --- | --- | --- | --- | --- | --- |
| Acc73 | 239027 | WG | Achefer | 2060 | Acc109 | 242272 | WG | Bure W | 2500 |
| Acc74 | 239030 | WG | Achefer | 2010 | Acc110 | 105007 | EG | Guzamn | 2430 |
| Acc75 | 239032 | WG | Achefer | 2000 | Acc111 | 216013 | EG | Guzamn | 2500 |
| Acc76 | 239034 | WG | Achefer | 2020 | Acc112 | 239028 | EG | Achefer | 2060 |
| Acc77 | 242308 | WG | Bd z | 1975 | Acc113 | 242248 | EG | Guzamn | 2450 |
| Acc78 | 242309 | WG | Bd z | 2000 | Acc114 | 242252 | EG | Guzamn | 2350 |
| Acc79 | 242310 | WG | Bd z | 1880 | Acc115 | 105008 | EG | Machakel |  |
| Acc80 | 239015 | WG | Bd z | 1910 | Acc116 | 105009 | EG | Machakel |  |
| Acc81 | 239016 | WG | Bd z | 1920 | Acc117 | 105010 | EG | Machakel |  |
| Acc82 | 239019 | WG | Bd z | 2000 | Acc118 | 105011 | EG | Machakel |  |
| Acc83 | 239046 | WG | Bure w | 2520 | Acc119 | 238996 | BD S | Bahir Dar | 2050 |
| Acc84 | 239051 | WG | Bure w | 2120 | Acc120 | 239035 | WG | Achefer | 2050 |
| Acc85 | 236620 | WG | Damot | 2110 | Acc121 | 239002 | WG | MerAwi | 2070 |
| Acc86 | 105006 | WG | Dembecha | 2430 | Acc122 | 105015 | EG | Machakel |  |
| Acc87 | 242263 | WG | Dembecha | 2380 | Acc123 | 105016 | EG | Machakel |  |
| Acc88 | 242264 | WG | Dembecha | 2430 | Acc124 | 105017 | EG | Machakel |  |
| Acc89 | 242265 | WG | Dembecha | 2450 | Acc125 | 242255 | EG | Machakel | 2200 |
| Acc90 | 242267 | WG | Dembecha | 2060 | Acc126 | 242256 | EG | Machakel | 2200 |
| Acc91 | 242269 | WG | Dembecha | 2010 | Acc127 | 242257 | EG | Machakel | 2120 |
| Acc92 | 242270 | WG | Dembecha | 2050 | Acc128 | 242258 | EG | Machakel | 2300 |
| Acc93 | 105001 | WG | Jabi | 2280 | Acc129 | 242260 | EG | Machakel | 2400 |
| Acc94 | 242303 | WG | Mecha | 1950 | Acc130 | 105004 | NG | Belesa | 1820 |
| Acc95 | 242304 | WG | Mecha | 1950 | Acc131 | 239012 | NG | G zuria | 1930 |
| Acc96 | 242305 | WG | Mecha | 2000 | Acc132 | 208464 | Awi | Dangela | 2100 |
| Acc97 | 242306 | WG | Mecha | 2010 | Acc133 | 239060 | NG | G zuria | 1900 |
| Acc98 | 242307 | WG | Mecha | 2010 | Acc134 | 208365 | GUR | Gumer |  |
| Acc99 | 236615 | WG | Mecha | 2000 | Acc135 | 225802 | N O | Dermalo | 2800 |
| Acc100 | 236616 | WG | Mecha | 2060 | Acc136 | 242320 | N O | Dermalo | 2800 |
| Acc101 | 238997 | WG | Mecha | 2060 | Acc137 | 207912 | Mk | Adwa |  |
| Acc102 | 238999 | WG | Mecha | 2050 | Acc138 | Local | WG | Dembecha |  |
| Acc103 | 239001 | WG | Mecha | 2050 | Acc139 | Local | Awi | Fagta |  |
| Acc104 | 239010 | WG | Mecha | 2050 | Acc140 | Local | WG | Achefer |  |
| Acc105 | 242249 | EG | Baso | 2300 | Acc141 | Local | WG | Mecha |  |
| Acc106 | 242250 | EG | Baso | 2310 | Acc142 | Local | SG | Dera |  |
| Acc107 | 242251 | EG | Baso | 2300 | Acc143 | Local | BD SP | Bd z |  |
| Acc108 | 242271 | WG | Bure w | 2450 | Acc144 | Sweet | Out-group* | | |

WG, West Gojam; EG, East Gojam; Bd Z, Bahir Dar zuria; Jabi, Jabi Tehnan; Baso, Baso Liben; Bure W, Bure Womberema; BD SP, Bahir Dar Special; NG, North Gondar; NO, North Omo; GUR, Gurage; MK, Mehakelegnaw; SG, South Gondar; Gm, Germany; G Zur, Gondar Zuria; Fagta, Fagta Lekoma; Locals, are farmers' cultivars cultivated in those mentioned areas; Out-group*, Kindly collected from Dr. Alemayehu Assefa (Adet research center, Ethiopia) and it is a blue sweet lupin (*Lupinus angustifolius*) genotype originally introduced from Germany.

S1 Table. (Continued)

| ACC no | EBI code | Zone | District | Altitude | ACC no | EBI code | Zone | District | Altitude |
| --- | --- | --- | --- | --- | --- | --- | --- | --- | --- |
| ACC145 | 228520 | Awi | Dangila |  | Acc180 | 239052 | WG | Dembecha | 2130 |
| Acc146 | 239000 | WG | Mecha | 2050 | Acc181 | 239053 | WG | Dembecha | 2150 |
| ACC147 | 242275 | Awi | Banja | 2550 | Acc182 | 239054 | WG | Dembecha | 2210 |
| ACC148 | 239014 | NG | G zuria | 1920 | Acc183 | 239055 | WG | Dembecha | 2160 |
| ACC149 | 242318 | SG | Dera | 2400 | Acc184 | 239056 | EG | Machakel | 2240 |
| ACC150 | 105012 | EG | Machakel |  | Acc185 | 239057 | EG | Machakel | 2380 |
| ACC151 | 105013 | EG | Machakel |  | Acc186_1 | 239058 | EG | Machakel | 2390 |
| ACC152 | 105014 | EG | Machakel |  | Aus1 | 239741 | Australia collections and donation | | |
| ACC153 | 229805 | Awi | Banja |  | Aus2 | 239742 |  |  |  |
| ACC154 | 239059 | EG | Gozamn | 2420 | Aus3 | 239743 |  |  |  |
| ACC155 | 242317 | SG | Dera | 2400 | Aus4 | 239744 |  |  |  |
| ACC156 | 238998 | WG | Mecha | 2060 | Aus5 | 239745 |  |  |  |
| ACC157 | 242312 | SG | Dera | 1960 | Aus6 | 239746 |  |  |  |
| ACC158 | 242289 | Awi | Fagta | 2375 | Aus7 | 239747 |  |  |  |
| ACC159 | 242285 | Awi | Guangua | 1790 | Aus8 | 239748 |  |  |  |
| ACC160 | 238995 | BD SP | Bahir Dar | 2020 | Aus9 | 239749 |  |  |  |
| ACC161 | 236618 | Awi | Dangila | 2100 | Aus10 | 239750 |  |  |  |
| ACC162 | 239031 | WG | Achefer | 2010 | Aus11 | 239751 |  |  |  |
| ACC163 | 216014 | EG | Baso | 2320 | Aus12 | 239752 |  |  |  |
| ACC164 | 216015 | EG | Machakel | 2280 | Aus13 | 239753 |  |  |  |
| ACC165 | 216016 | EG | Machakel | 2240 | Aus14 | 239754 |  |  |  |
| ACC166 | 239024 | BD SP | Bahir Dar | 1900 | Aus15 | 239755 |  |  |  |
| ACC167 | 239025 | BD SP | Bahir Dar | 1900 | Aus16 | 239756 |  |  |  |
| ACC168 | 239026 | BD SP | Bahir Dar | 1900 | Aus17 | 239757 |  |  |  |
| ACC169 | 239036 | WG | Achefer | 2000 | Aus18 | 239758 |  |  |  |
| ACC170 | 239037 | WG | Achefer | 2000 | Aus19 | 239759 |  |  |  |
| ACC171 | 239039 | WG | Achefer | 2080 | Aus20 | 239761 |  |  |  |
| ACC172 | 239040 | WG | Achefer | 2130 | Var01 | Variants identified while phenotyping the EBI collections | | | |
| ACC173 | 239041 | WG | Achefer | 2150 | Var02 |  |  |  |  |
| ACC174 | 239042 | WG | Achefer | 2120 | Var03 |  |  |  |  |
| ACC175 | 239043 | WG | Achefer | 2080 | Var04 |  |  |  |  |
| ACC176 | 239047 | WG | Bure W | 2660 | Var54_1 |  |  |  |  |
| ACC177 | 239048 | WG | Bure W | 2600 | Var54_2 |  |  |  |  |
| ACC178 | 239049 | WG | Bure W | 2480 | Acc186_2 | Variant from accession 186 | | | |
| ACC179 | 239050 | WG | Bure W | 2300 | Am | Out-group* | | | |

WG, West Gojam; EG, East Gojam; Bd Z, Bahir Dar zuria; Baso, Baso Liben; Bure W, Bure Womberema; BD S, Bahir Dar Special; NG, North Gondar; SG, South Gondar; G Zur, Gondar Zuria; Fagta, Fagta Lekoma; Out-group*, identified while phenotyping white lupin landraces accessed from EBI and found to be different species (*Lupinus* *mutabilis*).
