# Supplementary material for: Molecular genetic diversity and population structure of Ethiopian white lupin landraces: Implications for breeding and conservation
Source: PLoS One. 2017 Nov 30;12(11):e0188696. doi: 10.1371/journal.pone.0188696 (PMC5708786; doi:10.1371/journal.pone.0188696)
Supplement: S2 Table — (DOCX) [file pone.0188696.s002.docx]

S2 Table. List of SSR markers used for the study.

| Number | Marker  Name | Forward sequence | Reverse sequence | Source/Origin |
| --- | --- | --- | --- | --- |
| 1 | Lup125 | TGGTCCTATGTTGAAAGACC | CTGTACAACCAAATTACACCAG | Phan et al. (2007) & Raman et al. (2014) |
| 2 | Lup146 | ATTCCTCTTACCTAGTGCTTA | CCCTTGGGATTGATTGGTC | Phan et al. (2007) & Raman et al. (2014) |
| 3 | Lup197 | CTGGTTCTGTTCTTCAACTGTA | CCTATATGCCTTGGTCCAT | Phan et al. (2007) & Raman et al. (2014) |
| 4 | PT1-1 | CAAATGGAAAATCTTTGGAAGAG | CTAACATAAGACCTAAACATATGGAACAG | Phan et al. (2007) & Raman et al. (2014) |
| 5 | AnMtS13 | GCGACGTGCCCTCCAAGTCC | AGGACCCATGGAATCATTACCTCC | Phan et al. (2007) & Raman et al. (2014) |
| 6 | GLNA | GAATGGTGCTGGTGCTCACACA | TGGTGGTGTCTGCAATCATGGAAG | Phan et al. (2007) & Raman et al. (2014) |
| 7 | CHS9 | ATCCAGCCAACTGTGTTGAACAAAGCA | GATTTTGGTTGGCCCCATTCTTTTATAG | Phan et al. (2007) & Raman et al. (2014) |
| 8 | LSSR14 | GGTGACCCTCACCAGAACAT | GGTCCTTTGATGATGGTGCT | Phan et al. (2007) & Raman et al. (2014) |
| 9 | LSSR55 | CGAGGAAAGAGCAGTTTCAAG | CCTGGTAGTCCTTGGGTTCA | Phan et al. (2007) |
| 10 | LSSR9a | CCCCTGCTCCGAATATATGA | AAGCCCAATGATTGTTCTGG | Phan et al. (2007) |
| 11 | LSSR9b | CAAAGGATGGTTTCTTGTTAGGTC | CCTCGCACATTTCCTACCCA | Phan et al. (2007) |
| 12 | LSSR26a | TGCTCATGTTGCCAAGACTC | CGCAACAATCAGCTATAAGCC | Phan et al. (2007) |
| 13 | La1-EST01 | GCGCTAAATTTCTATCTTTTAACACT | CCCAACCATATTTTCCACCAAC | Phan et al. (2007) |
| 14 | PT1-2 | TTGTTGTCCCTGCAGAGATTTT | CAAACATAAGACCTAAACATATGGAACAG | Phan et al. (2007) |
| 15 | DSI | GAAGCCAAAAAGTATGAAGGGCCACGCAC | CATGGTGCATAAAACTCAACCAAGACATC | Phan et al. (2007) |
| 16 | Lup257 | GCTGGAGCAGATTATTGTGTA | CCTGGCAGACAGTTGCTT | Phan et al. (2007) |
