# Supplementary material for: Molecular genetic diversity and population structure of Ethiopian white lupin landraces: Implications for breeding and conservation
Source: PLoS One. 2017 Nov 30;12(11):e0188696. doi: 10.1371/journal.pone.0188696 (PMC5708786; doi:10.1371/journal.pone.0188696)
Supplement: S3 Table — (DOCX) [file pone.0188696.s003.docx]

S3 Table. The Evanno table output.

| K | Mean LnP(K) | Stdev LnP(K) | Ln'(K) | \|Ln''(K)\| | Delta K |
| --- | --- | --- | --- | --- | --- |
| 1 | -4072.3000 | 0.1225 | — | — | — |
| 2 | -3649.0200 | 2.3004 | 423.2800 | 164.8200 | 71.6473 |
| 3 | -3390.5600 | 40.7329 | 258.4600 | 294.8400 | 7.2384 |
| 4 | -3426.9400 | 310.6000 | -36.3800 | 287.2800 | 0.9249 |
| 5 | -3176.0400 | 205.4422 | 250.9000 | 105.0800 | 0.5115 |
| 6 | -3030.2200 | 49.3836 | 145.8200 | 51.1600 | 1.0360 |
| 7 | -2935.5600 | 29.7394 | 94.6600 | 77.3600 | 2.6013 |
| 8 | -2918.2600 | 15.8659 | 17.3000 | 9.0400 | 0.5698 |
| 9 | -2910.0000 | 71.0800 | 8.2600 | 58.7800 | 0.8270 |
| 10 | -2842.9600 | 44.8498 | 67.0400 | 13.3000 | 0.2965 |
| 11 | -2789.2200 | 42.4041 | 53.7400 | 46.6400 | 1.0999 |
| 12 | -2782.1200 | 19.0902 | 7.1000 | 7.2400 | 0.3793 |
| 13 | -2782.2600 | 26.4415 | -0.1400 | 4.7000 | 0.1778 |
| 14 | -2777.7000 | 44.1208 | 4.5600 | 2.2600 | 0.0512 |
| 15 | -2775.4000 | 70.9565 | 2.3000 | 108.3600 | 1.5271 |
| 16 | -2881.4600 | 128.1073 | -106.0600 | 47.6800 | 0.3722 |
| 17 | -2939.8400 | 234.4353 | -58.3800 | 208.1800 | 0.8880 |
| 18 | -2790.0400 | 78.8900 | 149.8000 | 209.0400 | 2.6498 |
| 19 | -2849.2800 | 183.8866 | -59.2400 | 44.0000 | 0.2393 |
| 20 | -2864.5200 | 136.9635 | -15.2400 | — | — |
